# An updated list of type material of Ephemeroptera Hyatt & Arms, 1890, deposited at the Zoological Museum of Hamburg (ZMH)

**DOI:** 10.3897/zookeys.607.9391

**Published:** 2016-07-26

**Authors:** Michel Sartori, Martin Kubiak, Hossein Rajaei

**Affiliations:** 1 Museum of zoology, Palais de Rumine, Place Riponne 6, CH-1005 Lausanne, Switzerland; 2 Zoologisches Museum, Centrum für Naturkunde (CeNak), Martin-Luther-King-Platz 3, 20146 Hamburg, Germany; 3 Staatliches Museum für Naturkunde Stuttgart, Rosenstein 1, 70191 Stuttgart, Germany

**Keywords:** Ephemeroptera, Hamburg, type specimens, Ulmer, ZMH

## Abstract

The type specimens of Ephemeroptera (Insecta) housed at the Zoological Museum of Hamburg (ZMH) are compiled in this document. The current nomenclature of all species is given. In total, Ephemeroptera type material of ZMH encompasses 161 species. Fifty-one holotypes and five lectotypes are present. Forty-one species are represented by syntypes, 85 by paratypes and five by paralectotypes. Material of two species (*Cinygma asiaticum* Ulmer, 1924 and *Pseudocloeon klapaleki* Müller-Liebenau, 1982) is missing. The present catalogue is an updated version of Weidner (1964a).

## Introduction

The Ephemeroptera collection of the Zoological Museum of Hamburg (ZMH) contains 433 species and approximately 4,500 specimens. In total, 161 species are represented by type material. In this sense, this is one of the largest collections with the highest number of type specimens of this insect order in Germany. Furthermore, the mayfly collection together with the caddisfly collection comprise some of the oldest voucher material of the entomological collection at the ZMH which was mainly collected at the end of the 19^th^ century and in the first decades of the 20^th^ century by the Hamburgian entomologist Dr. h.c. Georg Ulmer (1877–1963) (e.g., Weidner 1964b).

Georg Ulmer was born in Hamburg on 5 March 1877. He was the oldest son of seven siblings. After his father died in 1889, he decided to become a school teacher. During his qualification time in the early 1890s, he began collecting insects in the vicinity of Hamburg (Ulmer 1964). Initially, he started to compile a beetle collection but since he observed a mass emergence of mayflies at the river Fulda near Kassel in 1898, he started to focus his collecting efforts and taxonomic studies on primarily aquatic insect groups like mayflies or caddisflies (Ulmer 1964). In 1899, he finished his teacher training and started his career as a board school teacher in Hamburg for 32 years until 1934. Beside his profession, he studied voluntarily the faunistics, biology, systematics and taxonomy of Ephemeroptera (e.g., Ulmer 1904a, 1926, 1939) and Trichoptera (e.g., Ulmer 1905, 1951), and other freshwater invertebrates (e.g., Ulmer 1901, 1902a).

His first publication (Ulmer 1900) was followed by 175 scientific publications until his death on 15 January 1963 (Illies 1964). In the first years of his scientific career, he mainly studied the faunistics and taxonomy of caddisflies in the vicinity of Hamburg (e.g., Ulmer 1902b), or other localities in Germany (e.g. Ulmer 1904b, 1915). From the year 1905 on, he intensively started to study the material from foreign countries (including tropical regions), which has been lent to him by colleagues or other institutions (e.g., Ulmer 1905).

Besides the Trichoptera, Ulmer worked on mayflies (e.g., Ulmer 1908, 1909, 1912). Between 1904 and 1943, he published thirty research papers on the systematics and taxonomy of Ephemeroptera (Kimmins 1963; Illies 1964) with the description of 111 species (Weidner 1964a). His studies on mayflies resulted in the fundamental revision of the Southeast Asian mayfly fauna with the description of numerous species (Ulmer 1939).

During World War II (in 1943), the Zoological Museum Hamburg (that time located near the central railway station) was bombed and nearly all dry preserved material housed in the collections was destroyed and burned in the fire. Only material deposited in ethanol was transferred into the underground networks and survived the war (Weidner 1967). The valuable Ephemeroptera and Trichoptera collection of Georg Ulmer was kept in his private house outside of Hamburg. In 1964, the insect collection of Georg Ulmer was donated to the ZMH (Weidner 1964b) providing the majority of the recent voucher material of the ZMH in these two insect orders.

Georg Ulmer was an outstanding specialist and his comprehensive studies on the faunistics and taxonomy of various groups of freshwater invertebrates formed the foundation of the recent taxonomy and systematics of those groups, especially Ephemeroptera and Trichoptera.

In addition to Ulmer’s collection, the ZMH also stores primary type material described by other authors, such as Klapalek (1905a,b), Lestage (1930, 1935), Malzacher (1976, 2015, 2016), Müller-Liebenau (1971, 1974, 1980a, b, 1981), and Sartori (2008, 2014e). An interesting finding was the discovery of several paratypes of species described by Esben-Petersen (e.g. 1909, 1912a, b, 1913), which were not labelled as such but come from the type series, judging from the dates and places on labels. More information on these mayfly specialists can be found at http://www.ephemeroptera.de/inhaltsverz_deutsch/Galerie____/galerie____.html, a webpage maintained by Udo Jacob and Arne Haybach.

The only attempt, prior to this type catalogue, has been made by Weidner (1962, 1964a, 1977), who published a list of Ephemeroptera type material deposited in ZMH, without any taxonomic remarks. At that time, 121 species were mentioned. This catalogue is accessible online (https://www.cenak.uni-hamburg.de/sammlungen/zoologie/entomologie/typenkatalog.html).

Multiple subsequent taxonomic surveys utilizing a variety of different, high-resolution methods (morphological and molecular) led not only to the description of many new taxa, but also several supraspecific changes (e.g. Peters and Edmunds 1972; Puthz 1977; Braasch and Soldán 1986; Campbell and Suter 1988; Gillies 1990; Grant and Peters 1993; McCafferty and Waltz 1995; Lugo-Ortiz and McCafferty 1996, 1999; Sartori et al. 2008; Sartori 2014a, b, c.).

The catalogue that follows is arranged alphabetically by species name, followed by the original combination, author(s) name and year of publication. For each entry, an actual nomenclature (valid names or synonymy) is given. The kind of type and the metamorphic stage of specimens are abbreviated as indicated in the table header. The present study is a completely independent and new work; data reported by Weidner were not copied as a base for this catalogue. This study is entirely original work, based on the first author’s specimen-by-specimen examination of the ZMH collections from December 2013 to June 2014. Recent publications by Malzacher (2015, 2016) on Ulmer’s collection were also added.

**Table 1.** List of type material of Ephemeroptera Hyatt & Arms, 1890, deposited at the Zoological Museum of Hamburg, with the state and metamorphic stage of materials, current nomenclature, family assignment, related literature and complementary information. Abbreviations in this list are as follows: Type: HT= Holotype; LT= Lectotype; PT= Paratype(s); PLT= Paralectotype(s); ST = Syntypes. (state): (1) Specimen conserved in alcohol; (2) Pinned dry specimen; (3) Specimen or part of it mounted on microscopic slide. Stage: F= Female imago; M= Male imago; N= Nymph; SF= Female subimago; SM= Male subimago. Complementary information: a. Holotype wrongly designated by Weidner (1962, 1964a); b. Holotype designated by Ulmer on the label, not in the publication; c. Holotype apparently not in Stockholm (see http://www.nrm.se/download/18.1cb760b014a762ca801342e9/1421068753427/EPHEMEROPTERA_list.pdf); d. Specimen not labelled, but comes from the type series; e. Not type material! The types are the nymphs and the adults were only described in 1925; f. Only fore- and hindlegs in ZMH; g. Only fore wing in ZMH and remains in Naturkunde Museum, Berlin; h. Only foreleg; i. Only hind wing in ZMH and remains in Naturkunde Museum, Berlin; j. Only one forewing in ZMH and remains apparently not in Stockholm (http://www.nrm.se/download/18.1cb760b014a762ca801342e9/1421068753427/EPHEMEROPTERA_list.pdf); k. Wrongly considered by Ulmer, as a syn. of *Euchalcia bellieri* (Hagen).

Taxon	Original name	Author(s), year	Type (state)	Stage	Current nomenclature	Family	Literature & Complementary information
***alegrettae***	*Massartella alegrettae*	Ulmer, 1943	HT (2)	M	*Massartella alegrettae* Ulmer, 1943	Leptophlebiidae	(i.)
***ambiguum***	*Pseudocloeon ambiguum*	Müller-Liebenau, 1982	PT (1)	N	*Liebebiella ambigua* (Müller-Liebenau, 1982)	Baetidae	Waltz and McCafferty 1987
***ammophila***	*Oligoneuria ammophila*	Spieth, 1938	PT (1)	N	*Homoeoneuria ammophila* (Spieth, 1938)	Oligoneuriidae	Edmunds and Allen 1957
***anatolica***	*Rhithrogena anatolica*	Kazanci, 1985	PT (1)	M	*Rhithrogena anatolica* Kazanci, 1985	Heptageniidae	(d.)
***annandalei***	*Polymitarcys annandalei*	Chopra, 1927	ST (1)	F	*Ephoron annandalei* (Chopra, 1927)	Polymitarcyidae	Hubbard and Srivastava 1985
***apicatum***	*Cloeon apicatum*	Navás, 1933	ST (2)	M	*Cloeon navasi* van Bruggen, 1957	Baetidae	van Bruggen 1957; (d.)
***balcanicus***	*Metretopus balcanicus*	Ulmer, 1920	HT (3)	M	*Metreletus balcanicus* (Ulmer, 1920)	Ameletidae	Puthz 1977a; (f.)
***belgica***	*Torleya belgica*	Lestage, 1917	(PT) (2)	M, F	*Torleya major* (Klapalek, 1905a)	Ephemerellidae	(e.)
***bengalensis***	*Ecdyonurus bengalensis*	Ulmer, 1920	HT, PT (2, 3)	M, F, SM, SF	*Ecdyonurus bengalensis* Ulmer, 1920	Heptageniidae	(b.)
***berneri***	*Rheobaetis berneri*	Müller-Liebenau, 1974	HT (1)	N	*Heterocloeon berneri* (Müller-Liebenau, 1974)	Baetidae	McCafferty and Provonsha 1975
***bicorne***	*Centroptilum bicorne*	Ulmer, 1909	PT (1)	F	*Afroptilum bicorne* (Ulmer, 1909)	Baetidae	Gillies, 1990
***biobionicum***	*Deleatidium biobionicum*	Ulmer, 1938	PT (2)	F	*Meridialaris biobionica* (Ulmer, 1938)	Leptophlebiidae	Peters and Edmunds 1972; (g.)
***bishopi***	*Platybaetis bishopi*	Müller-Liebenau, 1980a	HT (1)	N	*Platybaetis bishopi* Müller-Liebenau, 1980	Baetidae	
***boettgeri***	*Pseudocloeon boettgeri*	Ulmer, 1924	ST (1, 2, 3)	M, F	*Pseudocloeon boettgeri* Ulmer, 1924	Baetidae	
***borneonia***	*Epeorella borneonia*	Ulmer, 1939	LT, PLT (1, 2)	M, F, SF	*Epeorella borneonia* Ulmer, 1939	Heptageniidae	Sartori 2014c; (a.)
***braueri***	*Hagenulodes braueri*	Ulmer, 1920	PT (1)	M	*Hagenulodes braueri* Ulmer, 1920	Leptophlebiidae	
***brenneriana***	*Rhithrogena brenneriana*	Klapalek, 1905a	PT (1)	M	*Rhithrogena alpestris* Eaton, 1885 (syn.)	Heptageniidae	Puthz 1975
***brunneum***	*Cloeon brunneum*	Esben-Petersen, 1909	ST (1, 2)	M, F	*Americabaetis peterseni* (Hubbard, 1974) (syn. obj.)	Baetidae	Lugo-Ortiz and McCafferty 1999
***caenoides***	*Neoephemeropsis caenoides*	Ulmer, 1939	LT, PLT (1, 3)	M, F, N	*Potamanthellus caenoides* (Ulmer, 1939)	Neoephemeridae	Bae and McCafferty 1998
***camerunense***	*Pseudocloeon camerunense*	Ulmer, 1920	ST (1, 3)	M, F	*Ophelmatostoma camerunense* (Ulmer, 1920)	Baetidae	Gillies et al. 1990
***canariensis***	*Baetis canariensis*	Müller-Liebenau, 1971	HT, PT (1)	M, N	*Baetis canariensis* Müller-Liebenau, 1971	Baetidae	
***cavum***	*Cinygma cavum*	Ulmer, 1927	PT (1)	M	*Cinygmula cava* (Ulmer, 1927)	Heptageniidae	Levanidova 1972
***chinensis***	*Baetis chinensis*	Ulmer, 1936	ST (1)	M, F	*Baetis chinensis* Ulmer, 1936	Baetidae	
***chinensis***	*Heptagenia chinensis*	Ulmer, 1920	ST (2)	M	*Heptagenia chinensis* Ulmer, 1920	Heptageniidae	(d.)
***chinensis***	*Siphluriscus chinensis*	Ulmer, 1920	PT (3)	M	*Siphluriscus chinensis* Ulmer, 1920	Siphluriscidae	(f.)
***corsicus***	*Ecdyonurus corsicus*	Esben-Petersen, 1912a	PT (2)	M	*Ecdyonurus corsicus* Esben-Petersen, 1912	Heptageniidae	Belfiore 1987; (k.)
***costaricanus***	*Leptohyphes costaricanus*	Ulmer, 1920	HT (2, 3)	F	*Tricorythodes costaricanus* (Ulmer, 1920)	Leptohyphidae	Bamgardner 2008
***crassinervis***	*Polyplocia crassinervis*	Ulmer, 1939	PT (2)	SM	*Polyplocia campylociella* Ulmer, 1939 (syn.)	Euthyplociidae	Demoulin 1953
***croaticus***	*Siphlonurus croaticus*	Ulmer, 1920	PT (1, 3)	M	*Siphlonurus croaticus* Ulmer, 1920	Siphlonuridae	
***curvata***	*Paraleptophlebia curvata*	Ulmer, 1927	PT (1, 3)	M	*Paraleptophlebia strandii* (Eaton, 1901) (syn.)	Leptophlebiidae	Kluge 2009
***deigma***	*Liebebiella deigma*	Waltz & McCafferty, 1987	HT, PT (1, 3)	N	*Liebebiella vera* (Müller-Liebenau, 1981) (syn.)	Baetidae	Kluge and Novikova 2011
***difficilum***	*Pseudocloeon difficilum*	Müller-Liebenau, 1982	PT (1, 3)	N	*Liebebiella difficila* (Müller-Liebenau, 1982)	Baetidae	Waltz and McCafferty 1987
***diptera***	*Hagenulopsis diptera*	Ulmer, 1920	ST (1)	M, SM, SF	*Hagenulopsis diptera* Ulmer, 1920	Leptophlebiidae	
***duporti***	*Ephemera duporti*	Lestage, 1921	PT (2)	M	*Ephemera duporti* Lestage, 1921	Ephemeridae	
***eatoni***	*Rhithrogena eatoni*	Esben-Petersen, 1912a	PT (2)	F	*Rhithrogena eatoni* Esben-Petersen, 1912	Heptageniidae	(d.)
***edmundsi***	*Platybaetis edmundsi*	Müller-Liebenau, 1980b	HT (1)	N	*Platybaetis edmundsi* Müller-Liebenau, 1980	Baetidae	
***ehrhardti***	*Thraulus ehrhardti*	Ulmer, 1920	ST (1, 2, 3)	M, F	*Needhamella ehrhardti* (Ulmer, 1920)	Leptophlebiidae	Dominguez and Flowers 1989
***eloisae***	*Derlethina eloisae*	Sartori, 2008	PT (1)	N	*Derlethina eloisae* Sartori, 2008	Teloganodidae	Sartori et al. 2008
***feuernborni***	*Pseudoligoneuria feuernborni*	Ulmer, 1939	ST (1, 3)	N	*Chromarcys magnifica* Navás, 1932 (syn.)	Oligoneuriidae	Kluge 2004
***flexus***	*Siphlurus flexus*	Clemens, 1913	PT (1)	M	*Siphloplecton basale* (Walker, 1853) (syn.)	Metretopodidae	McDunnough 1924
***fluviatile***	*Cloeon fluviatile*	Ulmer, 1920	PT (1, 3)	M, F	*Cloeon fluviatile* Ulmer, 1920	Baetidae	
***formosana***	*Ephemera formosana*	Ulmer, 1920	PT (2, 3)	M, F, SM, SF	*Ephemera formosana* Ulmer, 1920	Ephemeridae	
***formosanus***	*Chirotonetes formosanus*	Ulmer, 1912	ST (1, 2, 3)	M, SF	*Isonychia formosana* (Ulmer, 1912)	Isonychiidae	Ueno 1931
***fruhstorfferi***	*Massartella fruhstorfferi*	Ulmer, 1943	PT	SF	*Massartella brieni* (Lestage, 1924) (syn.)	Leptophlebiidae	Pescador and Peters 1990
***fuegiensis***	*Ameletus fuegiensis*	Lestage, 1935	HT (1, 3)	N	*Metamonius anceps* (Eaton, 1885) (syn.)	Nesameletidae	Mercado and Elliott 2004
***fulmeki***	*Acentrella fulmeki*	Ulmer, 1939	HT, PT (1, 3)	M, F	*Labiobaetis fulmeki* (Ulmer, 1939)	Baetidae	McCafferty and Waltz 1995
***fusca***	*Atalophlebia fusca*	Ulmer, 1920	HT (1)	M	*Koorrnonga fusca* (Ulmer, 1920)	Leptophlebiidae	Campbell and Suter 1988; (c.)
***gornostajevi***	*Epeorus gornostajevi*	Tshernova, 1981	PT (1)	M	*Epeorus gornostajevi* Tshernova, 1981	Heptageniidae	
***grandis***	*Chirotonetes grandis*	Ulmer, 1913	ST (1, 3)	M	*Isonychia grandis* (Ulmer, 1913)	Isonychiidae	Ulmer 1924
***guranica***	*Heptagenia guranica*	Belov, 1981	PT (1)	M	*Heptagenia guranica* Belov, 1981	Heptageniidae	
***haenschi***	*Euthyplocia haenschi*	Ulmer, 1942	PT (2)	M	*Euthyplocia haenschi* Ulmer, 1942	Euthyplociidae	
***horai***	*Potamanthellus horai*	Lestage, 1930	HT (2)	SM	*Potamanthellus amabilis* (Eaton, 1892) (syn.)	Neoephemeridae	Bae and McCafferty 1998
***hutanis***	*Dudgeodes hutanis*	Sartori, 2008	PT (1)	N	*Dudgeodes hutanis* Sartori, 2008	Teloganodidae	Sartori et al. 2008
***hyalinus***	*Ecdyonurus hyalinus*	Ulmer, 1912	ST (1, 2, 3)	M, F, SM, SF	*Afronurus hyalinus* (Ulmer, 1912)	Heptageniidae	Kang and Yang 1994
***insularis***	*Rhithrogena insularis*	Esben-Petersen, 1913	PT (2)	M, F	*Rhithrogena insularis* Esben-Petersen, 1913	Heptageniidae	(d.)
***irina***	*Cinygmula irina*	Tshernova & Belov, 1982	PT (1)	M	*Cinygmula irina* Tshernova & Belov, 1982	Heptageniidae	
***jacobsoni***	*Tricorythus jacobsoni*	Ulmer, 1913	ST (1, 3)	M, F	*Sparsorythus jacobsoni* (Ulmer, 1913)	Tricorythidae	Sroka and Soldán 2008; (a.)
***japonicus***	*Chirotonetes japonicus*	Ulmer, 1920	ST (1, 2, 3)	M, F	*Isonychia japonica* (Ulmer, 1920)	Isonychiidae	Ueno 1931; (a.)
***javanicus***	*Afronurus javanicus*	Ulmer, 1939	HT (1)	M	*Afronurus javanicus* Ulmer, 1939	Heptageniidae	
***javanicus***	*Baetis javanicus*	Ulmer, 1913	ST (1, 2, 3)	F	*Baetis javanicus* Ulmer, 1913	Baetidae	
***jorgenseni***	*Campsurus jorgenseni*	Esben-Petersen, 1912b	ST (1)	M, F	*Campsurus jorgenseni* Esben-Petersen, 1912	Polymitarcyidae	
***jorgenseni***	*Cloeon jorgenseni*	Esben-Petersen, 1909	ST (1, 3)	M, F	*Americabaetis jorgenseni* (Esben-Petersen, 1909)	Baetidae	Lugo-Ortiz and McCafferty 1999
***karnyi***	*Hagenulus karnyi*	Ulmer, 1939	ST (1, 3)	SM, SF	Choroterpes (Euthraulus) karnyi (Ulmer, 1939)	Leptophlebiidae	Peters and Edmunds 1970
***kraepelini***	*Pseudocloeon kraepelini*	Klapalek, 1905b	LT, PLT (1, 3)	M, SM	*Pseudocloeon kraepelini* Klapaleck, 1905	Baetidae	Waltz and McCafferty 1985
***krieghoffi***	*Chitonophora krieghoffi*	Ulmer, 1920	ST (2)	M, S	*Ephemerella mucronata* (Bengtsson, 1909) (syn.)	Ephemerellidae	Jacob 1974
***lacuscoerulei***	*Tasmanophlebia lacuscoerulei*	Tillyard, 1933	PT (1)	SF	*Tasmanophlebia lacuscoerulei* Tillyard, 1933	Oniscigastridae	(d.)
***laminatum***	*Deleatidum laminatum*	Ulmer, 1920	PT (3)	M	*Meridialaris laminata* (Ulmer, 1920)	Leptophlebiidae	Peters and Edmunds 1972; (h.)
***lamuriensis***	*Thalerosphyrus lamuriensis*	Sartori, 2014e	HT, PT (1, 3)	N	*Thalerosphyrus lamuriensis* Sartori, 2014	Heptageniidae	
***latifrons***	*Cinygmula latifrons*	Tshernova & Belov, 1982	PT (1)	M	*Cinygmula hirasana* (Imanishi, 1935) (syn.)	Heptageniidae	Kluge 1995
***latus***	*Tricorythus latus*	Ulmer, 1916	PT (1, 2, 3)	M	*Tricorythus latus* Ulmer, 1916	Tricorythidae	
***lestagei***	*Atalophlebiodes lestagei*	Ulmer, 1938	ST (1, 3)	N	*Meridialaris lestagei* (Ulmer, 1938)	Leptophlebiidae	Dominguez et al. 2006
***lieftincki***	*Heptagenia lieftincki*	Ulmer, 1939	HT, PT (1, 3)	M, F	*Compsoneuria lieftincki* (Ulmer, 1939)	Heptageniidae	Sartori 2014a
***lobatus***	*Ecdyonurus lobatus*	Ulmer, 1924	ST (1, 2, 3)	M, F, SF	*Afronurus lobatus* (Ulmer, 1924)	Heptageniidae	Mol 1987
***longilobata***	*Leptophlebia longilobata*	Tshernova, 1928a	PT (1, 3)	M	*Paraleptophlebia longilobata* (Tshernova, 1928)	Leptophlebiidae	Tiunova and Kluge 2016
***longus***	*Tricorythus longus*	Ulmer, 1916	PT (1, 2, 3)	M, F	*Tricorythus longus* Ulmer, 1916	Tricorythidae	
***lucida***	*Atalophlebia lucida*	Ulmer, 1920	HT (1)	M	*Thraulophlebia lucida* (Ulmer, 1920)	Leptophlebiidae	Demoulin 1955; (c.)
***macedonicus***	*Rhoenanthus macedonicus*	Ulmer, 1920	HT (2)	M	*Neoephemera maxima* (Joly, 1871)	Neoephemeridae	Bae and McCafferty 1998
***maculipennis***	*Thraulus maculipennis*	Ulmer, 1920	HT (1)	M	*Hermanella maculipennis* (Ulmer, 1920)	Leptophlebiidae	Dominguez and Flowers 1989
***magnificus***	*Rhoenanthus magnificus*	Ulmer, 1920	PT (1, 2, 3)	M	*Rhoenanthus magnificus* Ulmer, 1920	Potamanthidae	
***major***	*Choroterpides major*	Ulmer, 1939	HT, PT (1, 3)	M, N	*Dilatognathus major* (Ulmer, 1939)	Leptophlebiidae	Kluge 2012
***malaisei***	*Cinygma malaisei*	Ulmer, 1927	PT (1, 3)	M	*Cinygmula malesei* (Ulmer, 1927)	Heptageniidae	Levanidova 1972
***marginatus***	*Thraulus marginatus*	Ulmer, 1913	ST (1, 3)	M, F	Choroterpes (Euthraulus) marginatus (Ulmer, 1913)	Leptophlebiidae	Peters and Edmunds 1970
***media***	*Ephemera media*	Ulmer, 1936	ST (2)	M, F, SM, SF	*Ephemera media* Ulmer, 1936	Ephemeridae	(a.)
***media***	*Teloganopsis media*	Ulmer, 1939	HT, PT (1, 3)	M, N	*Teloganopsis media* Ulmer, 1939	Ephemerellidae	Ubero-Pascal and Sartori 2009; Sartori 2014f; (d.)
***melli***	*Thalerosphyrus melli*	Ulmer, 1926	PT (2, 3)	M, F	*Epeorus melli* (Ulmer, 1926)	Heptageniidae	Zhou et al. 2007
***mexicana***	*Heptagenia mexicana*	Ulmer, 1920	PT (3)	M	*Maccaffertium mexicanum* (Ulmer, 1920)	Heptageniidae	Wang and McCafferty 2004; (f.)
***mjoebergi***	*Euphyurus mjoebergi*	Ulmer, 1917	PT (1, 3)	M	*Ulmerophlebia mjoebergi* (Ulmer, 1917)	Leptophlebiidae	Demoulin 1955
***montium***	*Thraulus montium*	Ulmer, 1943	HT (2)	M	*Traverella montium* (Ulmer, 1943)	Leptophlebiidae	Allen 1973
***multus***	*Baetis multus*	Müller-Liebenau, 1984	PT (3)	N	*Labiobaetis multus* (Müller-Liebenau, 1984)	Baetidae	McCafferty and Waltz 1995
***nasuta***	*Heptagenia nasuta*	Ulmer, 1939	ST (1, 2)	M, F	*Trichogenia nasuta* (Ulmer, 1939)	Heptageniidae	Webb et al. 2006; (a)
***natans***	*Chankagenesia natans*	Buldowsky, 1935	PT (1)	M, F	*Chankagenesia natans* Buldowsky, 1935	Palingeniidae	
***necatii***	*Ecdyonurus necatii*	Kazanci, 1987	PT (1)	SM	*Electrogena necatii* (Kazanci, 1987)	Heptageniidae	Kazanci 2001
***necopinatus***	*Baetis necopinatus*	Müller-Liebenau, 1981	HT (1, 3)	M	*Labiobaetis necopinatus* (Müller-Liebenau, 1981)	Baetidae	McCafferty and Waltz 1995
***nigrescens***	*Baetis nigrescens*	Navás, 1932	ST (2)	M	*Baetis nigrescens* Navás, 1932	Baetidae	Müller-Liebenau 1971
***nigrescens***	*Tasmanophlebia nigrescens*	Tillyard, 1933	PT (1)	F, N	*Tasmanophlebia nigrescens* Tillyard, 1933	Oniscigastridae	(d.)
***nigropunctata***	*Caenis nigropunctata*	Klapalek, 1905b	ST (2, 3)	F	*Caenis nigropunctata* Klapalek, 1905	Caenidae	Malzacher 2015
***nigropunctatula***	*Caenis nigropunctatula*	Malzacher, 2015	HT, PT (1, 3)	M, F	*Caenis nigropunctatula* Malzacher, 2015	Caenidae	
***nitidum***	*Centroptilum nitidum*	Ulmer, 1916	ST (2)	F	*Bugilliesia nitida* (Ulmer, 1916)	Baetidae	Lugo-Ortiz and McCafferty 1996
***novatus***	*Baetis novatus*	Müller-Liebenau, 1981	HT (3)	N	*Baetis novatus* Müller-Liebenau, 1981	Baetidae	
***obscurum***	*Pseudocloeon obscurum*	Ulmer, 1913	ST (3)	M	*Pseudocloeon obscurum* Ulmer, 1913	Baetidae	Müller-Liebenau 1981
***olivascens***	*Baetis olivascens*	Ulmer, 1939	HT, PT (1, 3)	M, SM, SF	*Baetis olivascens* Ulmer, 1939	Baetidae	
***operosus***	*Baetis operosus*	Müller-Liebenau, 1984	PT (3)	N	*Labiobaetis operus* (Müller-Liebenau, 1984)	Baetidae	McCafferty and Waltz 1995
***orientale***	*Pseudocloeon orientale*	Müller-Liebenau, 1982	PT (1)	N	*Liebebiella orientale* (Müller-Liebenau, 1982)	Baetidae	Waltz and McCafferty 1987
***orientalis***	*Palingenia orientalis*	Chopra, 1927	PT (2)	M	*Palingenia orientalis* Chopra, 1927	Palingeniidae	
***orientalis***	*Raptobaetopus orientalis*	Müller-Liebenau, 1978	HT (3)	N	*Baetopus orientalis* (Müller-Liebenau, 1978)	Baetidae	Jacob 1991
***ornata***	*Rhithrogeniella ornata*	Ulmer, 1939	HT, PT (1, 3)	M, F	*Rhithrogeniella ornata* Ulmer, 1939	Heptageniidae	Sartori 2014d
***ornatum***	*Procloeon ornatum*	Tshernova, 1928b	LT (1, 3)	M	*Procloeon ornatum* Tshernova, 1928	Baetidae	Sowa 1975
***paradoxa***	*Anagenesia paradoxa*	Buldowsky, 1935	PT (1)	M, F	*Anagenesia paradoxa* Buldowsky, 1935	Palingeniidae	
***parvus***	*Ecdyonurus parvus*	Ulmer, 1912	ST (1, 2, 3)	M, F	*Rhithrogena parva* (Ulmer, 1912)	Heptageniidae	Sartori 2014b
***patagonica***	*Atalophlebia patagonica*	Lestage, 1931	HT (1, 3)	F	*Meridialaris patagonica* (Lestage, 1931)	Leptophlebiidae	Peters and Edmunds 1972
***pekinensis***	*Caenis pekinensis*	Malzacher, 2016	HT, PT (1,3)	N	*Caenis pekinensis* Malzacher, 2016	Caenidae	
***pekingensis***	*Baetis pekingensis*	Ulmer, 1936	HT, PT (1)	M, F, SM, SF	*Alainites pekingensis* (Ulmer, 1936)	Baetidae	Waltz et al. 1994
***peruvianus***	*Baetis peruvianus*	Ulmer, 1920	ST (2)	M, SM	*Andesiops peruvianus* (Ulmer, 1920)	Baetidae	Lugo-Ortiz and McCafferty 1999
***pescadori***	*Jubabaetis pescadori*	Müller-Liebenau, 1980b	HT (3)	N	*Jubabaetis pescadori* Müller-Liebenau, 1980	Baetidae	
***peterseni***	*Leptohyphes peterseni*	Ulmer, 1920	ST (1, 3)	M, F, SM, SF	*Leptohyphes peterseni* Ulmer, 1920	Leptohyphidae	
***petersi***	*Rheobaetis petersi*	Müller-Liebenau, 1974	HT (1)	M, N	*Heterocloeon petersi* (Müller-Liebenau, 1974)	Baetidae	McCafferty and Provonsha 1975
***philippinensis***	*Caenodes philippinensis*	Ulmer, 1924	ST (1, 2, 3)	M	*Caenis philippinensis* (Ulmer, 1924)	Caenidae	Kluge 1985
***pictipennis***	*Ephemera pictipennis*	Ulmer, 1924	ST (2)	M, F	*Ephemera pictipennis* Ulmer, 1924	Ephemeridae	
***powelli***	*Lachlania powelli*	Edmunds, 1951	PT (1)	M, F, N	*Lachlania saskatschevanensis* Ide, 1941 (syn.)	Oligoneuriidae	McCafferty 1996
***proba***	*Choroterpes proba*	Ulmer, 1939	ST (1, 3)	N	*Choroterpes proba* Ulmer, 1939	Leptophlebiidae	
***prominens***	*Habrophlebiodes prominens*	Ulmer, 1939	HT (1, 3)	M	*Habrophlebiodes prominens* Ulmer, 1939	Leptophlebiidae	
***pseudorhodani***	*Baetis pseudorhodani*	Müller-Liebenau, 1971	HT, PT (1)	M, N	*Baetis pseudorhodani* Müller-Liebenau, 1971	Baetidae	
***puigae***	*Teloganopsis puigae*	Ubero-Pascal & Sartori, 2009	PT (1)	N	*Teloganopsis puigae* Ubero-Pascal & Sartori, 2009	Ephemerellidae	
***pulcher***	*Afronurus pulcher*	Ulmer, 1930	PT (1)	SM, SF	*Afronurus collarti* (Navás, 1930) (syn.)	Heptageniidae	Demoulin 1956
***purpurata***	*Ephemera purpurata*	Ulmer, 1920	PT (2)	M	*Ephemera purpurata* Ulmer, 1920	Ephemeridae	
***ranauensis***	*Caenis ranauensis*	Malzacher, 2015	HT, PT (1, 3)	M, F	*Caenis ranauensis* Malzacher, 2015	Caenidae	
***rhenicola***	*Caenis rhenicola*	Malzacher, 1976	HT, PT (1)	M	*Caenis pusilla* Navás, 1913 (syn.)	Caenidae	Alba-Tercedor and Malzacher 1986
***rossicus***	*Ecdyonurus rossicus*	Tshernova, 1928b	PT (1)	M	*Kageronia fuscogrisea* (Retzius, 1783) (syn.)	Heptageniidae	Puthz 1977b
***sauteri***	*Ephemera sauteri*	Ulmer, 1912	PT (2)	M, F	*Ephemera sauteri* Ulmer, 1912	Ephemeridae	
***scotti***	*Caenis scotti*	Ulmer, 1930	PLT (1)	M, F	*Caenis scotti* Ulmer, 1930	Caenidae	
***seminiger***	*Simothraulus seminiger*	Ulmer, 1939	HT (1)	M	*Simothraulus seminiger* Ulmer, 1939	Leptophlebiidae	Grant and Peters 1993
***sexfasciata***	*Atalophlebia sexfasciata*	Ulmer, 1916	HT (1, 3)	M	*Atalomicria sexfasciata* (Ulmer, 1916)	Leptophlebiidae	Harker 1954; (j.)
***sikorai***	*Euthyplocia sikorai*	Vayssière, 1895	PT (1, 3)	N	*Proboscidoplocia sikorai* (Vayssière, 1895)	Euthyplociidae	Demoulin 1966
***sinensis***	*Iron sinensis*	Ulmer, 1926	PT (2, 3)	M	*Epeorus sinensis* (Ulmer, 1926)	Heptageniidae	Edmunds and Traver 1954
***soror***	*Baetis soror*	Ulmer, 1908	ST (1)	M, F	*Offadens soror* (Ulmer, 1908)	Baetidae	Lugo-Ortiz and McCafferty 1998
***sowai***	*Rhithrogena sowai*	Puthz, 1972	HT (2)	M	*Rhithrogena sowai* Puthz, 1972	Heptageniidae	
***sudanense***	*Centroptilum sudanense*	Ulmer, 1916	PT (1)	M, SM	*Bugilliesia sudanense* (Ulmer, 1916)	Baetidae	Lugo-Ortiz and McCafferty 1996
***sumatrana***	*Baetis sumatrana*	Ulmer, 1939	ST (1, 3)	M, F	*Baetis sumatrana* Ulmer, 1939	Baetidae	Müller-Liebenau 1981
***sumatranus***	*Ecdyonurus sumatranus*	Ulmer, 1939	HT (1, 3)	F	*Rhithrogena sumatrana* (Ulmer, 1939)	Heptageniidae	Sartori 2014b
***thienemanni***	*Compsoneuriella thienemanni*	Ulmer, 1939	LT, PLT (1, 2, 3)	M, F, N	*Compsoneuriella thienemanni* Ulmer, 1939	Heptageniidae	Sartori 2014a
***tibiale***	*Cinygma tibiale*	Ulmer, 1920	PT (1, 3)	M	*Rhithrogena tibiale* (Ulmer, 1920)	Heptageniidae	Tshernova and Belov 1982
***tibialis***	*Atopopus tibialis*	Ulmer, 1920	PT (2, 3)	M, F	*Atopopus tibialis* Ulmer, 1920	Heptageniidae	
***traverae***	*Rheobaetis traverae*	Müller-Liebenau, 1974	HT (1)	N	*Heterocloeon curiosum* (McDunnough, 1923) (syn.)	Baetidae	McCafferty and Provonsha 1975
***triangularis***	*Siphlonurus triangularis*	Clemens, 1915	PT (1)	M, F, N	*Siphlonurus quebecensis* (Provancher, 1878) (syn.)	Siphlonuridae	McDunnough 1925
***truncatus***	*Campsurus truncatus*	Ulmer, 1920	PT (2)	M, F	*Campsurus truncatus* Ulmer, 1920	Polymitarcyidae	
***ulmeri***	*Asionurus ulmeri*	Braasch & Soldán, 1986	HT, PT (1, 3)	N	*Asionurus ulmeri* Braasch & Soldán, 1986	Heptageniidae	
***ulmeri***	*Baetis ulmeri*	Müller-Liebenau, 1981	HT (1, 3)	M	*Labiobaetis ulmeri* (Müller-Liebenau, 1981)	Baetidae	McCafferty and Waltz 1995
***ulmeri***	*Behningia ulmeri*	Lestage, 1930	HT (1)	N	*Behningia ulmeri* Lestage, 1930	Behningiidae	
***ulmeri***	*Dudgeodes ulmeri*	Sartori, 2008	HT, PT (1, 3)	M, SM, F, N	*Dudgeodes ulmeri* Sartori, 2008	Teloganodidae	Sartori et al. 2008 Sartori 2014f
***ulmeri***	*Trichogenia ulmeri*	Braasch & Webb, 2006	HT, PT (1, 3)	N	*Trichogenia ulmeri* Braasch & Webb, 2006	Heptageniidae	
***ulmeriana***	*Caenis ulmeriana*	Malzacher, 2015	HT, PT (1, 3)	M, F	*Caenis ulmeriana* Malzacher, 2015	Caenidae	
***umbrata***	*Teloganella umbrata*	Ulmer, 1939	HT (1)	SF	*Teloganella umbrata* Ulmer, 1939	Teloganellidae	
***unguicularis***	*Euphyurus unguicularis*	Ulmer, 1917	PT (1)	M	*Austrophlebiodes unguicularis* (Ulmer, 1917)	Leptophlebiidae	Campbell and Suter 1988
***unguiculatus***	*Campsurus unguiculatus*	Ulmer, 1920	ST (2)	M	*Tortopsis unguiculatus* (Ulmer, 1920)	Polymitarcyidae	Molineri 2010
***ussingi***	*Rhithrogena ussingi*	Esben-Petersen, 1910	PT (2)	M	*Rhithrogena germanica* Eaton, 1885 (syn.)	Heptageniidae	Sowa 1971
***verum***	*Pseudocloeon verum*	Müller-Liebenau, 1982	PT (1, 3)	N	*Liebebiella vera* (Müller-Liebenau, 1981)	Baetidae	Waltz and McCafferty 1987
***virens***	*Cloeon virens*	Klapalek, 1905b	ST (2, 3)	M, F	*Cloeon virens* Klapalek, 1905	Baetidae	
***vitellinum***	*Centroptilum vitellinum*	Ulmer, 1939	HT (2)	M	*Centroptilum vitellinum* Ulmer, 1939	Baetidae	
***winckleri***	*Isonychia winckleri*	Ulmer, 1939	ST (2)	M, F, SF	*Isonychia winckleri* Ulmer, 1939	Isonychiidae	(a.)
***wui***	*Leptophlebia wui*	Ulmer, 1936	ST (1)	M, F	*Leptophlebia wui* Ulmer, 1936	Leptophlebiidae	
***wui***	*Caenis wui*	Malzacher, 2016	HT, PT (1,3)	N	*Caenis wui* Malzacher, 2016	Caenidae	
**Missing material**							
***asiaticum***	*Cinygma asiaticum*	Ulmer, 1924	ST (2)	M	*Incertae sedis*	Heptageniidae	Tshernova and Belov 1982
***klapaleki***	*Pseudocloeon klapaleki*	Müller-Liebenau, 1982	PT (1)	N	*Liebebiella klapaleki* (Müller-Liebenau, 1982)	Baetidae	Waltz and McCafferty 1987
